# Effects of Bedtime Periocular Warming on Sleep Status in Adult Female Subjects: A Pilot Study

**DOI:** 10.1155/2017/6419439

**Published:** 2017-10-08

**Authors:** Ichiro Sakamoto, Michihito Igaki, Tomohisa Ichiba, Masahiro Suzuki, Kenichi Kuriyama, Makoto Uchiyama

**Affiliations:** ^1^Personal Health Care Laboratory, Kao Corporation, Tokyo, Japan; ^2^Department of Psychiatry, Nihon University School of Medicine, Tokyo, Japan; ^3^Department of Psychiatry, Shiga University of Medical Science, Otsu, Shiga, Japan

## Abstract

Several studies have reported that suitable manipulation of human skin or body temperature can lead to improved sleep. To clarify the effect of skin warming on human sleep, 38 female subjects, who occasionally had difficulty with falling asleep, were studied. The participants underwent two experimental sessions, which were carried out in two consecutive follicular phases and randomly crossed over. The participants wore hot or sham eye masks in one 14-day session. The first half of each 14-day session was designated the baseline period (BL) without any interventions and the later half was designated the intervention period (INT), in which they wore either the hot or sham eye mask for 10 minutes at bedtime. All the participants were instructed to keep a sleep diary every morning for the BL and INT. The results showed that the hot eye mask was significantly preferred over the sham one with respect to comfort and that feelings of restfulness and being refreshed upon wakening in the morning were significantly better with the hot eye mask than with the sham. These results suggest that bedtime periocular warming has favorable effects on subjective well-being on awakening, possibly due to the sense of comfort experienced at bedtime.

## 1. Introduction

Stress, tension, and anxiety are major factors that disturb sleep [1], whereas relaxation is one of the most common interventions to improve sleep by alleviating such sleep-disturbing factors [2, 3]. Many relaxation techniques (e.g., progressive muscle relaxation, thought-stopping) have been reported to improve sleep quality, including those that reduce somatic and mental arousal [4, 5].

Recently, we have developed a disposable heat- and steam-generating (HSG) sheet that safely and easily warms the periocular, abdominal, or lumbar skin and reported that it is able to reduce sympathetic and increase parasympathetic nerve activity [6, 7], as well as enhancing alpha-band electroencephalogram activity [8], leading to potential relaxation of somatic arousal.

Other previous studies have shown that warming of hand or foot skin improves sleep quality in healthy elderly subjects with or without insomnia [9, 10], indicating that skin warming may have beneficial effects on sleep quality. However, the sleep-related effects of periocular skin warming have not yet been studied in detail.

To clarify the effects of periocular skin warming on subjective sleep quality, we focused on individuals with mild sleep problems. In the present single blind cross-over study employing subjects with occasional difficulty with initiating sleep, we applied our eye mask-type HSG sheet, or a sham sheet, at bedtime. For this purpose we included only female subjects, as it has been reported that mild sleep problems are more prevalent in women than in men [11, 12].

## 2. Materials and Methods

### 2.1. Subjects

Forty women, aged 23 to 39 (mean ± SD; 30.3 ± 4.9) years with regular menstrual cycles, who occasionally had difficulty with falling asleep but had no severe daytime consequences or marked sleep habit differences between workdays and holidays, were recruited through a clinical research organization. Women who habitually worked longer than 11 hours per day were excluded. None of the participants had any other sleep complaints or a history of sleep disorders or were taking sleep medication. Also none of them had engaged in shift work or had physical or psychiatric disorders or a history of them. All were nonsmokers, and did not habitually drink alcohol or caffeinated beverages before bedtime. Two women had declined to participate after receiving explanation of the experimental procedure. Ethical approval was obtained from the Ethics Committee of the Nihon University (approval number: 27-8), and written informed consent was obtained from all of the study subjects after they had received the detailed explanation of the experiment.

We assessed sleep status for one month prior to the experiment using the Japanese version of the Pittsburgh Sleep Quality Index (PSQI-J) [13] and finally included 35 participants whose PSQI-J score exceeded the cut-off point of 5.5. Of these 35 participants, 14 (40%) were married and 28 (80%) had an occupation.

### 2.2. Experimental Procedures

**Figure 1:**
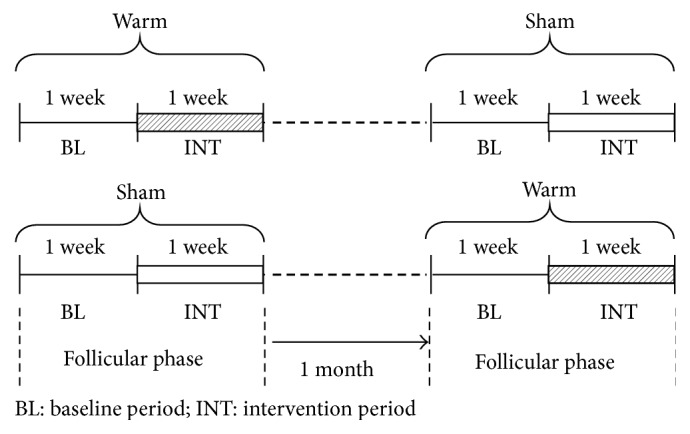
Schematic diagram of the experimental schedule.

The experimental schedule is shown in Figure 1. Each subject took part in two experimental sessions at her own home. During the experimental sessions, all of the participants were instructed to maintain their habitual sleep-wake schedule and habitual sleep environment. One session was set as a “warm session” and the other as a “sham session.” In the warm session, participants used a hot eye mask with the HSG, whereas in the sham session a sham eye mask without HSG was used. Two sessions were carried out in two consecutive follicular phases to control for possible effects of the menstrual cycle and randomly crossed over. The duration of each session was set at 2 weeks. The first half of the session was assigned as a baseline period (BL) without any interventions, and the later half as an intervention period (INT), in which the subjects were instructed to wear either type of eye mask (hot or sham) for 10 minutes when they went to bed. During the INT, the participants were instructed to abstain from alcohol or caffeinated beverages 6 hours before bedtime. All the participants kept a sleep diary every morning for the BL and INT.

### 2.3. Masks

The hot eye mask used in the warm session was made of nonwoven fabric and had a disposable HSG sheet, which provided moist heat through the chemical reaction of iron, water, and oxygen when its package was opened [14]. Our previous study showed that the hot eye mask warmed the periocular skin to 40°C within approximately 10 minutes [14]. The sham eye mask used in the sham session was made of a nonwoven fabric similar to that of the hot eye mask, but without the HSG. The masks covered both eyes and the periocular area, so that all outside vision was cut off. The eye masks were the prototype made by Kao Corporation for the present study.

### 2.4. Measures and Analysis

A 100 mm visual analogue scale (VAS) was used to assess subjective sleep status upon awakening, which consisted of five items related to a feeling of restfulness, feeling of being refreshed, sleep initiation, recovery from fatigue, and quality of sleep. Subjective sleep scores (sub-SS) in the morning were calculated by averaging the individual VAS scores during the BL and INT for each participant.

Participants were asked to keep a record of their lights-out time (LOT), sleep onset time (SOT), wakefulness after sleep onset (WASO), and rise time (RT) in the sleep diary every morning. Total sleep time (TST) was calculated by subtracting WASO from the time between SOT and RT. Time in bed (TIB) was calculated as the duration between LOT and RT. After completing an experimental session, participants were asked to describe which they felt was more preferable, the hot or the sham eye mask, from the viewpoint of comfort.

### 2.5. Statistical Analysis

Sub-SS, TST, and TIB were analyzed using two-way repeated measures ANOVA (session × period). Changes of subjective feelings with respect to condition preference were examined using two-tailed *t*-test. All statistical analyses were performed using IBM SPSS Statistics 20 (IBM, Chicago, IL). Significance was set at *P* < 0.05.

## 3. Results

Twenty-three participants (65.7%) reported that the hot eye mask was more comfortable than the sham one, whereas 7 (20.0%) preferred the sham eye mask (chi-squared test, *P* = 0.0001).

All of the participants adhered to the protocol. Sub-SS and subjective sleep time (TST and TIB) derived from the sleep diary are shown in Table 1. Two-way repeated measures ANOVA revealed no significant main effect of the period or session for the TST or TIB. However two-way repeated measures ANOVA demonstrated significant main effects of the period with respect to a feeling of restfulness (*F*(1, 34) = 10.4, *P* = 0.003), a feeling of being refreshed (*F*(1, 34) = 11.3, *P* = 0.002), sleep initiation (*F*(1, 34) = 8.9, *P* = 0.005), and quality of sleep (*F*(1, 34) = 12.2, *P* = 0.001) of the sub-SS. No effects of session were found in any items of the sub-SS. Significant period–session interactions were observed in the feeling of restfulness (*F*(1, 34) = 7.8, *P* = 0.008) and the feeling of being refreshed (*F*(1, 34) = 4.9, *P* = 0.034) of the sub-SS.

**Figure 2:**
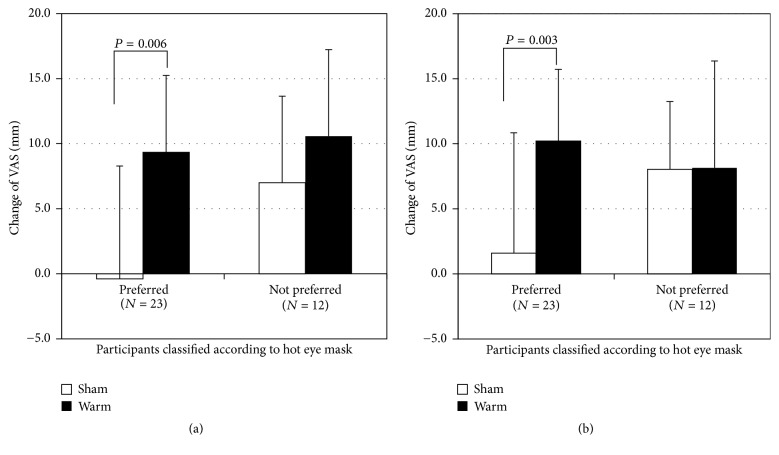
Changes in items of the sub-SS from BL to INT. Values are means ± SD. Participants who preferred the hot eye mask showed significant improvements in two items ((a) feeling of restfulness and (b) feeling of being refreshed) of the sub-SS after wearing the hot eye mask.

Among the participants who preferred the hot eye mask to the sham one, changes in the feelings of restfulness and being refreshed of the sub-SS from BL to INT were significantly greater in the warm session than in the sham session (two-tailed paired *t*-test, *P* < 0.01), whereas, among the participants who did not positively evaluate the hot eye mask, there were no significant differences in the changes of the sub-SS between the warm and sham sessions (Figure 2).

## 4. Discussion

The main positive outcome of the present study was that feeling of restfulness and that of being refreshed upon awakening were greater for periocular warming than for the sham intervention, while sleep initiation or total sleep time did not differ between the two conditions, suggesting that the improvements in the two subjective parameters related to sleep restoration upon awakening were not accompanied by subjective sleep changes. It has been reported that feelings of restfulness or refreshment upon awakening are disturbed by sleep difficulties due to various sleep disorders [15] and can be improved with appropriate therapeutic interventions [15]. The improvement of subjective feelings upon awakening in the present study did not seem to be attributable to prior subjective changes in nocturnal sleep, though a definitive or causal relationship between subjective feelings upon awakening and the sleep structure responsible for them has not yet been elucidated [15].

In the present study, favorable effects of periocular warming on feelings of restfulness and being refreshed were greater in the participants who preferred periocular warming to use of the sham mask. Many studies have shown that appropriate skin temperature manipulation [10, 16, 17], mainly warming, of various parts of the skin in humans can elicit a feeling of comfort and/or relaxation. A study using heart rate variability analysis has indicated that such favorable effects of periocular warming are attributable to acute activation of the parasympathetic nervous system [18]. Parasympathetic activation seems to be favorable in improving sleep quality because chronic insomnia is reported to be associated with abnormal sympathetic elevation [4], and several established treatments for insomnia may work through parasympathetic activation [4]. In the present study, however, there appeared to be no subjective changes in sleep after periocular warming, at least in terms of the subjective sleep parameters we evaluated. Given that the preference for periocular warming over use of the sham mask was influenced by a feeling of relaxation related to parasympathetic activation, as demonstrated in the previous study [18], it can be postulated that parasympathetic tone associated with periocular warming before sleep in the present study might have contributed to a more comfortable psychological state at bedtime, thus influencing the participants' subjective feelings upon awakening. As other mechanisms, it could be considered that periocular warming before sleep enhances slow wave sleep (SWS) through the thermoregulatory system and enhancements of SWS affects endocrine or immunity system, causing subjective sleep changes.

There were several limitations to the present study. First, the research results were based on subjective evaluations made by the participants, and physiological indices were not measured quantitatively. In the next study, it will be necessary to use objective evaluations such as EEG. Second, the experiment was carried out under home conditions. Though the participants were asked to maintain their habitual life and sleep environment, potential confounding factors that could have affected the participants' sleep status, such as room temperature, humidity, illumination, and noise level, were not completely controlled for. Further investigation will be necessary to clarify the effects of periocular warming under various environmental conditions. Third, since the participants were able to distinguish the warm condition from the sham condition easily in the present study, the results may have been confounded by the feelings experienced when wearing the eye mask. Fourth, the sample sizes of the participants allocated by job or marital status were small and uneven. Although we attempted to exclude subjects who were overworked or who had improper sleep habits, occupation and marital status might have been confounding factors impacting sleep quality. Fifth, the hot eye mask used in the present study warmed the periocular skin to 40°C within approximately 10 minutes. The duration of 10 minutes warming was set up based on the previous study [14] measured parasympathetic activation and so on. However, further investigation on applying the hot eye mask with the duration of more than 10 minutes warming will be necessary to clarify the relation of the duration of warming and sleep quality.

## 5. Conclusions

Our results suggest that periocular warming at bedtime was effective for improving subjective sleep parameters such as feelings of restfulness and being refreshed in the morning. Our present findings, and the mechanisms responsible, need to be confirmed in a more sophisticated experimental setting such as polysomnographic evaluation.

## Figures and Tables

**Table 1:** Subjective sleep scores in the morning and subjective sleep time derived from the sleep diary.

Measurements	Sham session		Warm session		ANOVA		
	BL	INT	BL	INT	Main effect		Interaction
					Period	Session	
Subjective sleep score (mm)							
Feeling of restfulness	51.3 ± 13.1	53.5 ± 12.9	47.9 ± 12.0	57.7 ± 13.4	*∗∗*	n.s.	*∗∗*
Feeling of being refreshed	46.6 ± 13.7	50.4 ± 14.0	45.2 ± 13.3	54.7 ± 14.7	*∗∗*	n.s.	*∗*
Sleep initiation	54.7 ± 14.2	59.5 ± 17.2	52.9 ± 15.5	61.1 ± 17.9	*∗∗*	n.s.	n.s.
Recovery from fatigue	48.7 ± 11.2	51.7 ± 12.8	47.3 ± 12.6	52.2 ± 14.0	n.s.	n.s.	n.s.
Quality of sleep	50.0 ± 13.5	55.1 ± 13.0	48.0 ± 10.7	55.0 ± 12.7	*∗∗*	n.s.	n.s.
Subjective sleep time (min)							
Total sleep time (TST)	387.2 ± 53.8	391.5 ± 67.2	378.6 ± 54.2	394.4 ± 47.2	n.s.	n.s.	n.s.
Time in bed (TIB)	426.9 ± 54.0	420.4 ± 56.5	410.0 ± 54.4	414.5 ± 49.3	n.s.	n.s.	n.s.

BL, baseline period; INT, intervention period; ANOVA, analysis of variance. Values are means ± SD. *n* = 35. ^*∗*^*P* < 0.05; ^*∗∗*^*P* < 0.01.
